# Evaluation of Patients under Investigation for MERS-CoV Infection, United States, January 2013–October 2014

**DOI:** 10.3201/eid2107.141888

**Published:** 2015-07

**Authors:** Eileen Schneider, Christina Chommanard, Jessica Rudd, Brett Whitaker, Luis Lowe, Susan I. Gerber

**Affiliations:** Centers for Disease Control and Prevention, Atlanta, Georgia, USA

**Keywords:** Middle East respiratory syndrome, MERS, coronavirus, MERS-CoV, patients under investigation, PUI, surveillance, United States, viruses

## Abstract

Middle East respiratory syndrome (MERS) cases continue to be reported from the Middle East. Evaluation and testing of patients under investigation (PUIs) for MERS are recommended. In 2013–2014, two imported cases were detected among 490 US PUIs. Continued awareness is needed for early case detection and implementation of infection control measures.

Middle East respiratory syndrome coronavirus (MERS-CoV) infection was first reported in September 2012 in a patient with fatal pneumonia in Saudi Arabia (*1*). Subsequent investigation showed that earlier MERS-CoV infection had occurred in Jordan in April 2012 among a cluster of patients with pneumonia (*2*,*3*). As of February 5, 2015, the World Health Organization had reported 971 laboratory-confirmed cases worldwide and at least 356 related deaths (*4*). All known reported cases have been linked directly or indirectly to the Middle East region; most have been reported by Saudi Arabia and the United Arab Emirates. Typically, the initial symptoms for MERS patients seeking medical care are fever, chills, cough, shortness of breath, and myalgia. These symptoms often progress to severe lower respiratory tract infection, which may require mechanical ventilation and intensive care (*5*,*6*). Several asymptomatic or mild MERS cases have been reported (*7*), particularly in healthy young adults. Little is known about transmission routes, virus shedding, risk factors, and animal reservoirs, although bats and camels have been implicated in transmission and as reservoirs (*8*,*9*). Clusters of human-to-human transmission have been associated with household and health care settings (*2*,*3*,*5*).

Using World Health Organization guidelines and definitions (*4*), CDC developed guidance for evaluating a patient under investigation (PUI) for MERS-CoV infection, collecting specimens, conducting laboratory testing, and managing infection control (http://www.cdc.gov/coronavirus/mers/index.html). The PUI guidance was created to assist health care providers determine which patients should be considered for MERS-CoV evaluation and testing. To inform state and local health departments of the basic demographic and clinical characteristics of PUIs and on assay use, we summarized the descriptive analysis of PUIs in the United States.

## The Study

In October 2012, CDC developed real-time reverse transcription PCR (rRT-PCR) assays for detection of MERS-CoV (*10*). CDC initially performed the testing, but on June 5, 2013, a Food and Drug Administration–issued Emergency Use Authorization allowed for assay deployment in a kit to laboratories through the Laboratory Response Network. As of March 12, 2015, a total of 47 states and the District of Columbia had MERS-CoV testing capability. The assay kit is intended for detection of MERS-CoV RNA in respiratory, serum, and stool samples. CDC also developed serologic tests for detecting MERS-CoV antibodies; these tests have been used by CDC since the summer of 2013. Because MERS-CoV is an emerging pathogen, CDC guidelines and guidance regarding PUI characteristics are periodically updated as new MERS-CoV information and risk factors are identified. CDC recommends evaluating and testing PUIs for MERS-CoV and for other common respiratory pathogens. 

On January 1, 2013, CDC began collecting data on PUIs for MERS-CoV infection. Health care providers for persons suspected of having MERS were to contact their state or local health department for consultation and to arrange for MERS-CoV testing, if indicated. PUIs were reported to CDC through state and local health departments by using the single-page PUI short form, which contains no personal identifiers (*11*). Since its implementation, the short form has been revised 3 times to reflect modifications to the PUI guidance. The short form collects information on basic demographic data, symptoms, disease severity, hospitalization, travel history, risk factors, and laboratory test results at the time of MERS-CoV testing. Follow-up data collection on missing information was not routinely performed. At least 370 (76%) PUIs met the guidance characteristics for PUI for MERS. The remaining 120 (24%) PUIs had incomplete clinical or travel data; the most common missing information was pneumonia data for persons with respiratory symptoms and a recent travel history. The short form was sent electronically to CDC by secure fax or email. Data collected on the short form was entered into a CDC database by using Microsoft Access (Microsoft Corporation, Redmond, WA, USA). SAS version 9.3 (SAS Institute, Cary, NC, USA) was used for data analysis.

**Figure 1:**
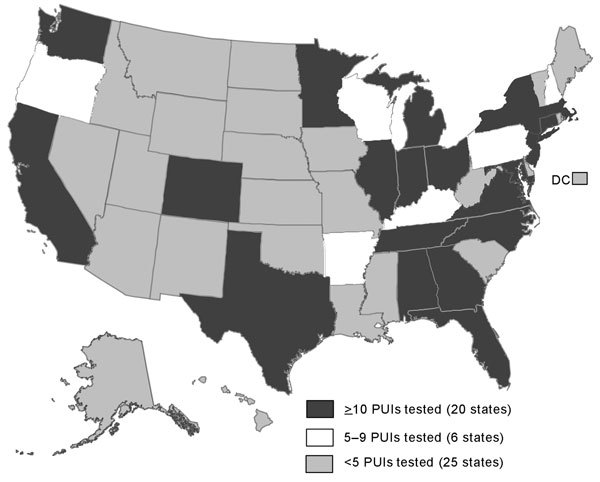
Number of PUIs tested for MERS-CoV (N = 490), by state, United States, January 1, 2013–October 31, 2014. DC, District of Columbia; PUIs, patients under investigation.

**Figure 2:**
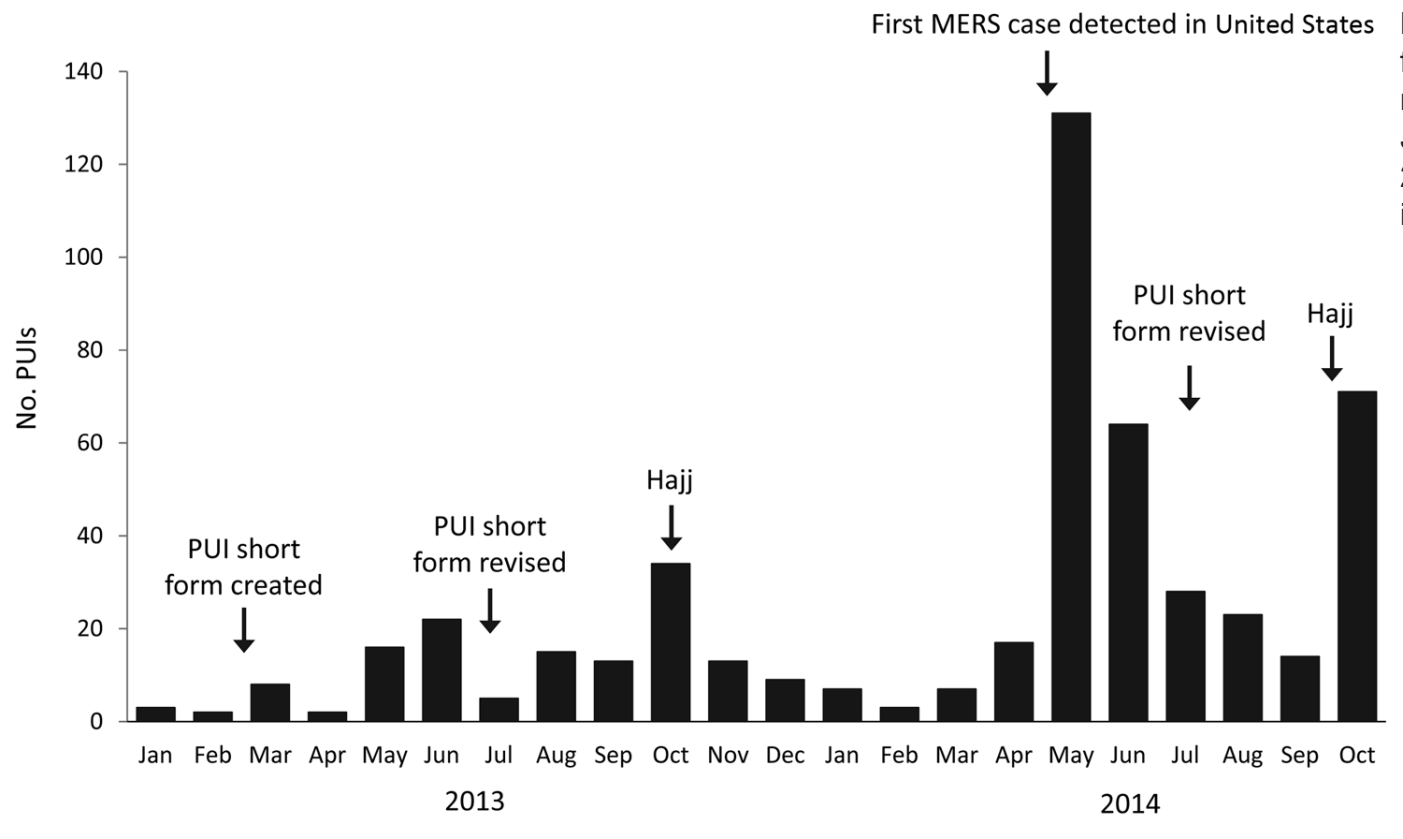
Number of PUIs tested for MERS-CoV (N = 490), by month reported, United States, January 1, 2013–October 31, 2014. PUIs, patients under investigation.

During January 1, 2013–October 31, 2014, a total of 490 PUIs were reported to CDC from 45 states and the District of Columbia (Figures 1, 2; Table 1). Of the 490 PUIs, 381 (78%) reported traveling from the Arabian Peninsula or neighboring countries to the United States within 14 days before illness onset (Table 2). A total of 113 (23%) PUIs also reported having close contact with a recently ill traveler from the Arabian Peninsula or neighboring countries within 14 days of symptom onset; the most common contacts were with persons from Saudi Arabia (55/113 [49%]), United Arab Emirates (10/113 [9%]), and Qatar (9/113 [8%]). Non-US residents accounted for 113 (23%) of the PUIs.

**Table 1:** Characteristics of 490 PUIs for MERS-CoV, United States, January 1, 2013–October 31, 2014*

Characteristic	Value
Sex, M/F	296 (60.4)/186 (38.0)
Median age, y (range)	48 (0.3–89)
Symptom	
Cough	393(80.2)
Fever	388 (79.2)
Shortness of breath	220 (44.9)
Chills†	153 (35.1)
Myalgia†	140 (32.1)
Sore throat	134 (27.4)
Headache†	106 (24.3)
Diarrhea†	58 (13.3)
Abdominal pain†	34 (7.8)
Hospitalized	292 (59.6)
Intensive care unit	112 (38.4)
Mechanical ventilation	61 (20.9)
Clinical finding	
Pneumonia‡	236 (48.2)
Acute respiratory distress syndrome‡	48 (9.8)
Renal failure	22 (4.5)
Died	11 (2.2)
Underlying condition†	
Immunosuppression	55 (12.6)
Diabetes	40 (9.2)
Hypertension	27 (6.2)
Cardiac disease	23 (5.3)
Asthma	20 (4.6)
Chronic pulmonary disease	11 (2.5)
Hyperlipidemia	9 (2.1)
Pregnant	8 (1.8)
Renal disease	7 (1.6)
Other	12 (2.8)
Specific job classification†	
Health care worker	35 (8.0)
US military	9 (2.1)
MERS-CoV rRT-PCR testing	
Specimen type	
Upper respiratory	414 (84.5)
Lower respiratory	242 (49.4)
Serum	235 (48)
Stool	40 (8.2)
MERS-CoV positive	2 (0.4)
Serologic testing for MERS-CoV	
Tested	67 (13.7)
MERS-CoV positive	2 (0.4)
Other pathogens detected§	
Influenza A virus	41 (8.4)
Rhinovirus/enterovirus	37 (7.6)
Influenza B virus	13 (2.7)
* Streptococcus pneumoniae*	11 (2.2)
Human metapneumovirus	6 (1.2)
Adenovirus	5 (1.0)
Coronavirus	4 (0.8)
Respiratory syncytial virus	4 (0.8)
Parainfluenzavirus	3 (0.6)
* Chlamydophila pneumoniae*	2 (0.4)
* Legionella pneumoniae*	2 (0.4)
Other	16 (3.3)

*Data are no. (%) unless otherwise specified. MERS-CoV, Middle East respiratory syndrome coronavirus; PUI, patient under investigation; rRT-PCR, real-time reverse transcription PCR. †Included only in 2 most recent versions of PUI short form (N = 436). ‡Forty-one PUIs had pneumonia and acute respiratory distress syndrome. §Etiologic pathogen not reported for 359 (73%) PUIs; >1 pathogen identified for some PUIs.

**Table 2:** Countries from which 381 PUIs for MERS-CoV infection had traveled within 14 days of symptom onset, United States January 1, 2013–October 31, 2014*

Country	No. (%)
Saudi Arabia	189 (49.6)
United Arab Emirates	60 (15.7)
Israel	45 (11.8)
Jordan	34 (8.9)
Qatar	27 (7.1)
Kuwait	22 (5.8)
Egypt	12 (3.1)
Bahrain	10 (2.6)
Oman	9 (2.4)
Iran	8 (2.1)
Iraq	8 (2.1)
Lebanon	8 (2.1)
Turkey	8 (2.1)
Pakistan	6 (1.6)
Palestinian Territories	6 (1.6)
Yemen	6 (1.6)
Other†	13 (3.4)

*Patients may have been in >1 country. PUI, patient under investigation. †Azerbaijan (1); Afghanistan (2); Bangladesh (1); Greece (1); India (2); Indonesia (2); Kenya (1); Morocco (1); Somalia (1); Syria (1).

The most commonly reported symptoms were cough, fever, and shortness of breath (Table 1). A total of 292 (60%) PUIs were hospitalized, of whom 112 (38%) were admitted to the intensive care unit and 61 (21%) required mechanical ventilation. The most commonly reported underlying conditions among PUIs were immunosuppression and diabetes. Eleven (2%) PUIs died. 

In total, 488 PUIs tested negative for MERS-CoV by rRT-PCR, serologic assay, or both. In May 2014, two PUIs tested positive for MERS-CoV by serologic assay and rRT-PCR in serum and respiratory samples, including lower respiratory tract specimens. These 2 patients were health care providers in whom respiratory symptoms had developed within 14 days of travel from Saudi Arabia; both cases were identified as imported MERS (*12*,*13*). Neither patient required mechanical ventilation. 

The most commonly detected pathogens among the 490 PUIs were influenza A virus and rhinovirus/enterovirus; however, for 359 PUIs (73%), other pathogen testing was not performed or detected pathogens were not reported (Table 1). Timely reporting of PUIs to CDC may have influenced reporting of pending non–MERS-CoV etiologic pathogen test results.

## Conclusions

Currently in the United States, the preferred method for detecting MERS in PUIs with recent symptom onset is to test lower respiratory, naso-oropharyngeal, and serum specimens by using the CDC rRT-PCR assay. For PUIs in whom symptom onset occurred >2 weeks before specimen collection, testing using the CDC MERS-CoV serologic assay on a single serum specimen is recommended. CDC also recommends testing for common respiratory pathogens (e.g., influenza virus), but identification of a respiratory pathogen should not preclude MERS-CoV testing (*14*). The PUI guidance serves to help health care providers and state health departments identify patients for evaluation and testing for MERS-CoV infection; however, because we are still learning about the natural history of MERS-CoV, it is reasonable to consider testing for MERS-CoV even when some PUI characteristics are not present, especially in the presence of strong clinical or epidemiologic judgment for MERS-CoV. During the evaluation process for MERS-CoV infection, appropriate infection control measures should be instituted as soon as possible for hospitalized and nonhospitalized PUIs (*15*).

The 2 US cases of imported MERS were detected in health care providers from Saudi Arabia. These cases prompted a CDC guidance update recommending evaluation and testing of persons with less severe respiratory illness who had strong epidemiologic risk factors, particularly heath care exposure, for MERS-CoV infection. Occupation, recent travel history, recent visit to a health care facility, and contact will ill persons should be determined when evaluating persons with respiratory illness. As testing increases, especially serologic testing, additional MERS cases, including mildly symptomatic cases and cases among younger persons are being identified. These cases highlight the range of severity of MERS-CoV infection and the need to consider testing persons with a compatible travel history who may not match the clinical profile of the initially described case-patients. CDC plans to revise MERS-CoV guidance as needed.
